# The Pacific Northwest National Laboratory library of bacterial and archaeal proteomic biodiversity

**DOI:** 10.1038/sdata.2015.41

**Published:** 2015-08-18

**Authors:** Samuel H. Payne, Matthew E. Monroe, Christopher C. Overall, Gary R. Kiebel, Michael Degan, Bryson C. Gibbons, Grant M. Fujimoto, Samuel O. Purvine, Joshua N. Adkins, Mary S. Lipton, Richard D. Smith

**Affiliations:** 1 Biological Sciences Division, Pacific Northwest National Laboratory, Richland, Washington 99354, USA; 2 Environmental Molecular Science Laboratory, Pacific Northwest National Laboratory, Richland, Washington 99354, USA

**Keywords:** Environmental microbiology, Pathogens, Proteome informatics, Proteomics

## Abstract

This Data Descriptor announces the submission to public repositories of the PNNL Biodiversity Library, a large collection of global proteomics data for 112 bacterial and archaeal organisms. The data comprises 35,162 tandem mass spectrometry (MS/MS) datasets from ~10 years of research. All data has been searched, annotated and organized in a consistent manner to promote reuse by the community. Protein identifications were cross-referenced with KEGG functional annotations which allows for pathway oriented investigation. We present the data as a freely available community resource. A variety of data re-use options are described for computational modelling, proteomics assay design and bioengineering. Instrument data and analysis files are available at ProteomeXchange via the MassIVE partner repository under the identifiers PXD001860 and MSV000079053.

## Background & Summary

Global measurements of -omic molecular data (genome, transcriptome, proteome, metabolome, etc.) are changing the way we research and think about biological systems. Computational biology research, which attempts to identify novel biological phenomena using these large-scale global measurements, depends on publically available data for training and testing new algorithms. Repositories like GEO^1^ were vital to the development of robust computational methods for analyzing microarray and other genomics technologies. Therefore, depositing complementary proteomics data for a large number of organisms is a similarly valuable public resource.

Researchers at the Pacific Northwest National Laboratory have participated in hundreds of collaborative projects that have involved mass spectrometry-based proteomic analysis of more than 300 species or distinct environmental communities. A portion of this data has been freely available through our website (omics.pnl.gov) for almost a decade, while metadata is maintained by our in-house LIMS systems^2^. In addition to the numerous project specific publications, meta-analyses of this massive corpus have advanced both computational algorithms^3–5^ and biological discovery^6–8^. The size of the library, however, has precluded broad distribution due to a lack of public repositories large enough to host the data. Recently, the ProteomeXchange^9^ repository system enabled accommodation of significantly larger data volumes.

**Figure 1:**
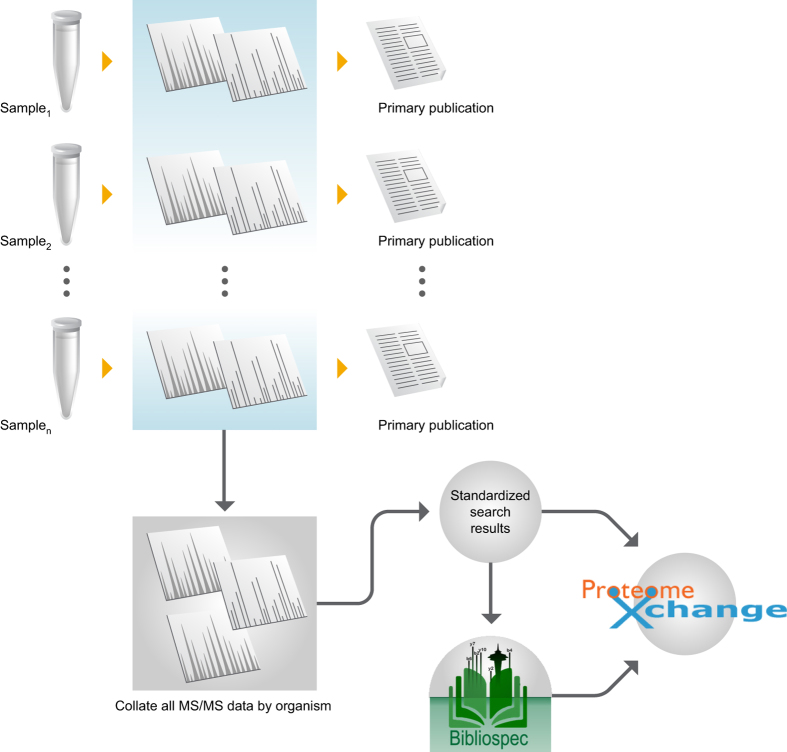
Workflow for library creation. Biological samples are used to create MS/MS data as part of an experiment and primary publication. All of this data is stored on our servers for re-analysis. Historical data was collated by organism and researched for release in the Biodiversity Library.

The purpose of this Data Descriptor is to announce the deposition of proteomics data from 112 microbial organisms representing 15 phyla into public 3rd party repositories (Table 1 (available online only)). All the data has been prepared, parsed and organized in a uniform manner to facilitate analysis and reuse (Fig. 1). The combined data deposited is 13 TB (compressed) from 35,162 mass spectrometry files and their associated analysis files. In total, the library contains >70 million spectra identified at q<0.0001, with 3 million peptides from 230,000 proteins. The median number of observed proteins per organism is 2154, or roughly half of the annotated proteins in the proteome. By releasing this data, we hope to promote open science. In this manuscript, we describe a variety of re-uses for mass spectrometry, algorithmic computation and basic biology.

**Figure 2:**
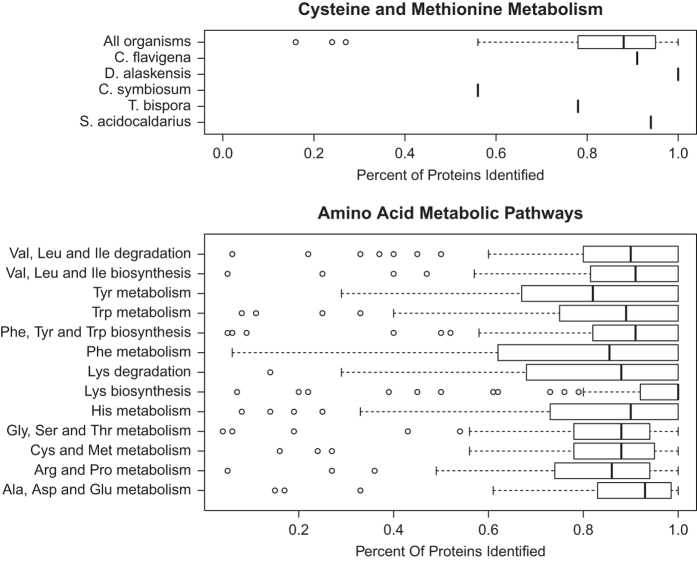
Kegg pathway coverage. (top) Using the pathway classifications provided by KEGG, we can determine how many annotated proteins were identified in mass spectrometry data. The cysteine and methionine metabolism pathway is provided as an example. For each organism, we calculate the percentage of identified proteins. *C. flavigena* has 23 proteins in the pathway, 21 of which were observed (91%). The box plot shows average coverage of the 103 organisms that KEGG has annotated. Circles depict outliers. (bottom) Pathway coverage for all 13 amino acid metabolic pathways is shown.

As part of the analysis, we have cross referenced protein identifications to KEGG functional annotation where possible. Nine of the 112 organisms are not processed by KEGG, and therefore were excluded from this additional analysis. When viewing the Library as a whole, annotated biological pathways are broadly covered by the identified proteins. For example, the reference ‘cysteine and methionine metabolism pathway’ as defined by KEGG consists of 81 orthologous genes participating in 73 reactions. As expected, not all orthologs are annotated in every genome, e.g., *Cellulomonas flavigena* has only 23 of the 81 genes. By searching all MS/MS data with standard RefSeq databases, we can easily identify that 21 of the 23 *Cellulomonas* genes were observed in MS/MS data, or 91%. When considering all organisms in the Library, the median coverage of the cysteine and methionine metabolism pathway is 89%. A summary of the coverage of every KEGG pathway for each organism is presented in Supplementary Table 1. Using KEGG pathway categories, we determined the median coverage of all functionally classified proteins (Fig. 2). For example, in all 13 pathways for amino acid metabolism, the median coverage across the entire library is 89%. This high coverage is seen for most KEGG pathway categories: 82% for lipid metabolism, 83% for vitamin and cofactor metabolism, etc.

## Methods

As the library encompasses 35,162 mass spectrometry files from 10+ years of research, it is impossible to fully describe the evolving and diverse protocols for experimental sample preparation or data acquisition. In Supplementary Table 2, we have provided data from our LIMS system^2^ about each sample data file (called a dataset). Below is a set of descriptions that represent a large fraction of the methods applied to generate the released datasets.

Either an established or optimized protein extraction protocol was applied to each sample^7^. In brief, a typical experimental approach included global (total), insoluble, and soluble protein extractions from lysed cell cultures that were then washed and suspended in 100 mM NH_4_HCO_3_, pH 8.4 buffer.

Global protein extracts were denatured and reduced by adding urea, thiourea, and dithiothreitol (DTT) followed by incubation at ~60 °C for ~30 min. Following incubation, the global protein samples were diluted to reduce salt concentration and then proteolytic digested, at 37 °C for ~4 h, using sequencing grade trypsin (Roche, Indianapolis, IN) at a ratio of 1 unit per 50 units of protein (1 unit=~1 μg of protein). Following incubation, digested samples were desalted using an appropriately sized C-18 SPE column (Supelco, St Louis, MO) and a vacuum manifold. The collected peptides were concentrated to a final volume ranging from 50 to 100 μl and measured using the BCA assay (Pierce Chemical Co., Rockfort, IL) according to the manufacturer's instructions.

Insoluble protein extracts were produced by ultracentrifuging the cell lysate at 4 °C and 100,000 rpm for 10 min. The resulting supernatant that contained soluble proteins was separated from the pellet and retained for digestion as previously described for the global extraction. The pellet was washed by suspending it in 100 mM NH_4_HCO_3_, pH 7.8, using mild sonication and then ultracentrifuged at 100,000 rpm for 5 min, again at 4 °C. Following centrifugation, the pellet was resuspended in a solubilizing solution that contained urea, thiourea, 1% CHAPS in 50 mM NH_4_HCO_3_, pH 7.8. An aliquot of 50 mM DTT solution was also added to final concentration of 5 mM. The insoluble protein sample was then incubated and digested as described above with the exception that a 50 mM NH_4_HCO_3_, pH 7.8 buffer was used for the dilution step. Following proteolytic digestion, the pH of the sample was slowly lowered to <4.0 by adding small volumes (1 to 2 μl) of 20% formic acid. Removal of salts and detergent was performed using either an appropriately sized strong cation exchange (SCX) or solid phase extraction column (Supelco, St Louis, MO) and vacuum manifold. Peptides were then concentrated and their concentration measured as described above.

The HPLCs used to run the samples were built in-house utilizing various commercial pumps, valves, and auto samplers, all of which were coordinated by a custom software package called LCMSnet. The data sets analyzed for this paper were run using LC columns that were 75 μm inner diameter, and either 30 or 65 cm in length. These LC columns were packed in house with Phenomenex Jupiter C18 3 μm porous beads. The flow rate was 300 nl/min. Mobile phase A is 0.1% formic acid in water and mobile phase B is 0.1% formic acid in acetonitrile. The 100 min gradient was delivered by starting at 5% mobile phase B and advancing to 8, 12, 35, 60, and 75% at times (in minutes) 2, 20, 75, 97, 100 respectively. Typically 2.5 μg of peptides were loaded to the head of the column or to a trapping column. Although operating conditions varied by capabilities of each instrument, typical conditions for each are as follows. The LTQ was run in data-dependent MS-MS mode, selecting the top 10 parent ions from each survey scan. The LTQ-Orbitrap and the Velos-Orbitrap instruments were typically set to have a high resolution survey scan of 60,000 resolution followed by the top 6 or 10 data-dependent MS-MS scans, respectively. Because of the diversity of data sets presented in this work, this is not a comprehensive list of conditions. Instrumentation details can be found in the raw data files (.RAW or.mzML).

### Code availability

Software used in the generation of this project is largely third party software as described in the Data Records section, i.e., MSGF+ and Bibliospec. The only remaining software was to link protein identifications to KEGG functional assignments. This was done via custom parsing of the files and cross-referencing the KEGG database. This code is trivially reproducible.

## Data Records

To maximize the utility and ease of access, the data described in this publication have been uploaded to the ProteomeXchange^9^ with accession PXD001860 via MassIVE (Data Citation 1). On MassIVE (identifier MSV000079053), each organism’s data is located in a separate folder, with both raw and processed data as described below. Data is organized around a tandem mass spectrometry file that represents one run of the instrument on a biological sample. In our terminology this is called a dataset. Each dataset has the following associated files.

### Mass spectrometry data

Each dataset is available in the original vendor format and the community standard open format mzML^10^. These files contain the raw mass spectra. Mass spectrometry data is a combination of MS and MS/MS data showing both the detection of all analytes at a particular time in chromatography (MS data) and the fragmentation of a particular analyte (MS/MS data). See the review by Aebersold and Mann for a basic primer of proteomic mass spectrometry data^11^.

### Peptide identifications

Each dataset is associated with a file describing the peptides that were identified via the spectra. This file was created using the MSGF+ algorithm^12^ version v9979. All 35,162 datasets were analyzed with a consistent set of parameters. Searches included oxidized methionine as an optional post-translational modification, and specified partial trypsin specificity. For experiments that utilized iodoacetimide as an alkylation agent, the static modification (C+57) was also added. Precursor and fragment mass tolerances were set according to the resolving power of the mass analyzer. The output of MSGF+ is stored in the community standard mzIdentML format^13^, which describes the peptide/spectrum match (PSM), search parameters and scoring details.

The one caveat for peptide identification was that three organisms did not have a RefSeq proteome set derived from a publically available genome sequence. *Escherichia coli* RK4353 did not have a sequence genome at NCBI, so we used the relative BW2952 strain. *Cyanothece* strain ATCC51472 also lacks a sequence at NCBI; we substituted strain 8801. *Thiocapsa marina* DSM_5653T lacks a RefSeq genome; the Genbank submission was used instead.

### Metadata

Data acquired at PNNL has been tracked using an in-house LIMS system since 2000. Each dataset is recorded with a variety of details including: acquisition date and time, instrument, chromatography details, organism, etc. These metadata are presented in Supplementary Table 2 with this publication.

### Spectrum library

A spectrum library is a condensed collection of annotated tandem mass spectra. In addition to serving as an efficient storage format for very large datasets, these libraries are also utilized for annotating new datasets^14,15^. With this deposition, we created a spectrum library for each microbial organism using Bibliospec^16^. Peptide/spectrum matches were filtered for high quality matches (MSGF+’s q-value <0.0001). When viewed in aggregate, the 112 organisms had 70,455,991 spectra passing this cutoff (with 1951 false hits and an estimated FDR of 2e-5). This strict filtering is necessary to control false-positives when creating very large libraries. The libraries, stored as.blib files, are also available on the MassIVE repository.

## Technical Validation

When releasing the Library, we took a conservative stance on spectral quality. Considering the large number of spectra, even a 1% false-positive rate would mean polluting the resource with nearly one million false-positive spectral identifications. Moreover, a well-known problem in proteomics is that aggregating numerous datasets leads to the inflation of false-positives when considering the entire group. This is especially true when rolling results up to a peptide or protein level as many true spectra are associated with a single true protein, whereas false proteins are typically represented by very few false-positive spectra identifications. The primary method to reduce false-positive peptide and protein identifications is to be more stringent on spectrum quality.

**Figure 3:**
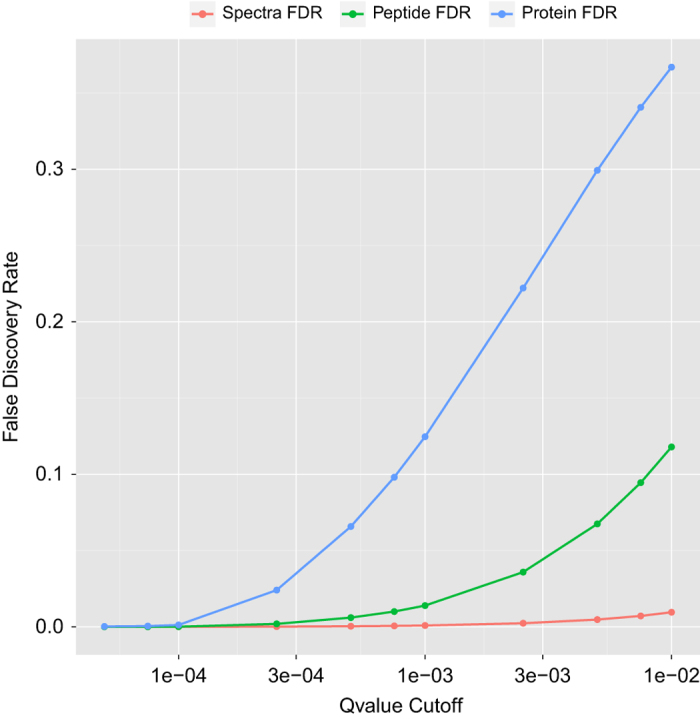
False Discovery Rate. Due the large nature of the Library, the false-discovery rate of the aggregated data can inflate significantly, especially when rolled up to protein and peptide level. Data is shown for the FDR of the entire Library when using a specified qvalue cutoff of PSMs from the MSGF+ results. When using a loose PSM filter of qvalue<0.01, the protein and peptide FDR rates are unacceptably high. We choose the cutoff qvalue<0.0001, which produces high data quality at spectra, peptide and protein levels.

When aggregating 35,162 datasets into the Library, using a typical qvalue cutoff of 0.01 on each individual dataset was insufficient to ensure high quality of the library as a whole (Fig. 3). Although the spectral false discovery was indeed 1%, the protein level false discovery was an astonishing 37%. We applied a qvalue cutoff of 0.0001, or two orders of magnitude more stringent than common practice. In this filtering process, 23 million true-positive spectra are removed. Although this may seem overly conservative, the more stringent filter also removed 600,000 false-positive peptides and 200,000 false-positive protein identifications. This allowed for a permissible false-discovery rate at spectrum, peptide, and protein levels (0.00002, 0.00009 and 0.001 respectively).

## Usage Notes

**Figure 4:**
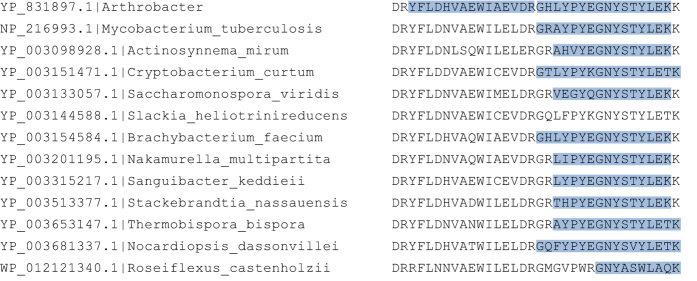
Peptide observation across taxa. This is a multiple sequence alignment of a section of an ABC transporter (accessions and organism given), with observed peptides from the PNNL Biodiversity Library in blue. For simplicity, we displayed sequences from the Actinobacteria phylum, with *Roseiflexus* as an out group. The right side of the alignment shows consistent discovery in proteomics data across the phylum and in the out group. The left side of the alignment is only observed in the proteomics data for *Arthrobacter* sp. FB24.

Our purpose in depositing such a large corpus of data is to promote reuse and open science. The richness of the PNNL Biodiversity Library is seen in both the breadth and depth of coverage for proteins and phylogeny. Besides sheer size, a unique feature of the Library is the pairs of spectra that come from similar peptides; one million peptides in the Library are one mutation away from another peptide (edit distance=1). These pairs originate from orthologues, where the proteins share significant sequence identity (Fig. 4). Indeed, 21,721 peptides have four or more ‘one mutation’ neighbours. This vast web of sequence related spectra can be productively mined for a wide variety of bioinformatics and fundamental mass spectrometry research.

### Ion fragmentation

Exploring the fundamentals of fragmentation is typically done working with purified peptides in low throughput^17,18^. With the Biodiversity library, however, pairs of related spectra could easily be mined to understand the effect of residue changes on the intensity of fragment ions. For example, there are 2,854 peptides where sequences only differ in that an alanine residue is changed to a serine residue. Additionally, many peptides are repeatedly identified. Indeed 53,828 peptides have over 200 spectra. Replicate spectra for a peptide are often used in understanding and modelling fragmentation patterns. However, in the library we note that 30,672 peptides with over 200 spectra are from conserved regions of proteins found in multiple organisms. Thus they contain distinct background and noise in the MS/MS spectra, aiding in the identification of novel fragment peaks.

### Proteotypic peptides

Computational prediction of which peptides are discoverable in experimental conditions is a valuable tool in proteomics workflows^19^. Such machine learning efforts will undoubtedly improve with the 3 million peptides provided by the PNNL Biodiversity Library. Yet the related sequences mentioned above provide a truly distinct perspective on peptide observability. Several important features of orthology can be utilized to improve the quality of machine learning predictions. First, as seen in Fig. 4, there are regions of a protein sequence which are fundamentally observable. In many orthologs spanning a large phylogeny, these regions are consistently observed. The sequence variation present in these regions can be leveraged to identify the physiochemical factors that govern mass spectrometry identification. Also seen are regions that are rarely observed. These could provide valuable negative training data for machine learning approaches.

### Library search of MS/MS data

Spectrum annotation via library search is both faster and more sensitive than database search algorithms^20^. Due to a lack of data, library search has previously not been practical except for the most commonly used model systems (e.g., human and yeast). Since the Biodiversity Library contains data for nearly every model system, including numerous environmentally and medically relevant microbes, peptide identification via spectrum library matching becomes an attractive alternative to database searching.

### Novel scoring functions

Bioinformatics algorithms to identify peptides from mass spectrometry data are constantly being developed and refined. For these, having free access to a large pool of training data is essential^12,21–23^. With data presented on different classes of instruments and multiple fragmentation modalities, the PNNL Biodiversity Library is an ideal source of data to test new scoring functions.

### Unidentified spectra

Another application that we envision is the investigation of unidentified or unattributed spectra. With tens of thousands of LC-MS/MS data sets, there are literally hundreds of millions of fragmentation spectra for which there is not a confident identification using the current search tool and parameters. Of those unidentified species, many are fragmented in multiple data sets; spectrum averaging or other methods could be utilized to obtain a confident identification.

### Novel post-translational modifications

For simplicity and sensitivity, only the most common post-translational modification (oxidized methionine) was included in the database search parameters. However, numerous post-translational modifications are observable in proteomics mass spectrometry^24^. Some modifications are rare, and therefore not commonly included in database searches. We recently uncovered a novel PTM switch in *Salmonella* for S-thiolation^25^ and believe that many such unexpected post-translational modifications exist. Identifying which observed PTMs are functionally relevant is a difficult task, but observing it across different taxa and showing evolutionary conservation provides a valuable filter for high-priority targets^26,27^.

### Proteogenomics

The process of using peptides from mass spectrometry to assist genome annotation, or proteogenomics, has been very successful in identifying both false-negative omissions in a genome’s protein list, and also false-positives. To date most of the work in this area has been focused on a single genome, or a group of closely related genomes^4,28,29^. With the Biodiversity Library, one can now attempt to leverage identifications across an entire phylum, or perhaps the entire tree of life.

## Additional Information

**How to cite this article:** Payne, S. H. *et al.* The Pacific Northwest National Laboratory library of bacterial and archaeal proteomic biodiversity. *Sci. Data* 2:150041 doi: 10.1038/sdata.2015.41 (2015).

## Supplementary Material



Supplementary Table 1

Supplementary Table 2

## Figures and Tables

**Table 1:** Overview of the organisms included in the PNNL Biodiversity Library

**Organism Name**	**# datasets**	**Proteins**	**Peptides**	**Spectra**	**False Spectra**
Acidiphilium_cryptum_JF-5	40	1454	7306	123,944	0
Actinosynnema_mirum_DSM_43827	85	2083	13975	80,974	0
Anabaena_variabilis	341	3324	35814	456,600	13
Anaeromyxobacter_dehalogenans	20	1250	7685	54,092	0
Anaplasma_phagocytophilium	129	690	3066	8,363	0
Arthrobacter_sp_FB24	353	3279	46122	663,861	29
Bacillus_anthracis_Ames	20	849	6487	36,014	0
Bacillus_anthracis_Sterne	158	2506	26165	152,339	0
Bacillus_subtilis_168	242	2435	39415	709,179	0
Bartonella_henselae_Houston-1	160	1245	36347	508,803	1
Borrelia_burdorferi_B31	104	877	19060	193,743	1
Brachybacterium_faecium_DSM_4810	78	2088	29248	121,914	0
Burkholderia_mallei	189	1970	33824	270,609	2
Candidatus_chloracidobacterium_thermophilum	111	563	2360	6,037	7
Caulobacter_crescentus_CB15	1016	2971	63773	2,320,212	41
Cellulomonas_flavigena_DSM_20109	103	2382	38033	175,148	0
Cenarchaeum_symbiosum	108	358	757	4,172	0
Chlorobaculum_tepidum_WT	94	1584	26561	137,150	0
Chloroflexus_aurantiacus	225	2736	44200	323,036	16
Clostridium_thermocellum	408	2110	61618	1,140,251	5
Cryptobacterium_curtum_DSM_15641	78	1115	17888	75,903	0
Cyanobacterium_synechocystis_PCC6803	279	2251	27351	344,473	0
Cyanothece_sp_ATCC51142	1404	3309	57219	1,879,239	0
Cyanothece_strain_ATCC51472	93	2146	20702	134,879	0
Cyanothece_strain_PCC7424	109	2182	20763	91,389	0
Cyanothece_strain_PCC7425	104	2590	27429	114,498	0
Cyanothece_strain_PCC7822	166	3230	37493	336,052	0
Cyanothece_strain_PCC8801	90	2399	23313	145,714	0
Cyanothece_strain_PCC8802	81	679	3958	22,785	0
Dehalococcoides_ethenogenes	437	1025	25378	262,644	0
Deinococcus_radiodurans_R1	368	2212	43409	1,002,931	0
Delta_proteobacterium_NaphS2	158	2210	13007	94,378	0
Desulfovibrio_desulfuricans_G20	463	2564	48057	916,183	28
Desulfovibrio_sp_ND132	92	2573	42717	225,546	0
Desulfovibrio_vulgaris_Hildenborough	218	2397	36518	451,518	0
Dethiosulfovibrio_peptidovorans_DSM_11002	78	1778	25287	95,940	0
Ehrlichia_chaffeensis	127	532	4045	12,545	0
Enterobacter_cloacae_SCF1	58	1793	19194	235,721	0
Escherichia_coli_BL21	191	1564	20792	190,645	0
Escherichia_coli_K-12	3925	3324	122114	11,592,291	461
Escherichia_coli_RK4353	31	1766	17089	53,334	0
Fibrobacter_succinogenes_S85	122	1505	17408	265,219	0
Geobacter_bemidjiensis_Bem_T	780	2566	33313	682,897	0
Geobacter_metallireducens_GS-15	155	2455	27442	245,691	0
Geobacter_sulfurreducens_PCA	1122	2770	45298	2,597,915	0
Geobacter_uraniumreducens	287	2779	42812	401,586	0
Haloferax_volcanii	34	1463	9502	56,669	0
Halogeometricum_borinquense_DSM_11551	83	2216	18776	56,225	0
Halorhabdus_utahensis_DSM_12940	84	2082	22987	89,820	0
Heliobacterium_modesticaldum	77	1549	22105	107,100	0
Kineococcus_radiotolerans_SRS30216	192	2742	38694	480,705	0
Kosmotoga_olearia_TBF_19-5-1	25	878	7219	29,080	0
Methanosarcina_barkeri	89	1852	24889	116,224	0
Methanospirillum_hungatei_JF-1	78	1722	23041	60,300	0
Methylophilales_HTCC2181	61	1172	20615	104,603	2
Mycobacterium_tuberculosis	647	2714	35206	1,296,018	7
Nakamurella_multipartita_DSM_44233	78	2171	19782	57,582	0
Nocardiopsis_dassonvillei_DSM_43111	80	1965	16507	76,404	0
Novosphingobium_aromaticivorans_F199	23	1563	12783	75,850	1
Opitutaceae_bacterium_TAV2	125	2229	20659	292,644	0
Pelagibacter_ubique_HTC1062	426	1201	30536	1,294,945	58
Pelobacter_carbinolicus_DSM_2380	22	830	4168	19,078	0
Prochlorococcus	121	1285	19310	158,225	0
Pseudomonas_aerunginosa	40	3054	24670	109,854	1
Pseudomonas_fluorescens_PfO-1	397	3437	44819	535,883	0
Pseudonocardia_sp	48	188	448	1,406	0
Ralstonia_pickettii	194			5,786	0
Rhodobacter_capsulatus_SB1003	495	2761	58894	1,316,844	76
Rhodobacter_sphaeroides_2.4.1	1639	3362	72977	3,982,596	47
Rhodopseudomonas_palustris	587	2743	25366	247,373	0
Roseiflexus_castenholzii	76	2537	28414	91,063	0
Saccharomonospora_viridis_DSM_43017	78	1918	15458	39,384	0
Salmonella_typhi_TY2	499	2729	50292	9,832,722	385
Salmonella_typhimurium_ATCC_14028	3701	3779	91496	1,555,617	28
Salmonella_typhimurium_LT2	823	3214	62399	1,051,565	3
Sanguibacter_keddieii_DSM_10542	78	2213	20489	89,427	0
Shewanella_amazonensis_SB2B	114	2079	27295	469,397	32
Shewanella_baltica_OS155	631	2988	47725	1,874,615	112
Shewanella_baltica_OS185	190	2853	41613	395,867	4
Shewanella_baltica_OS195	65	2193	23560	175,237	5
Shewanella_baltica_OS223	75	2158	22391	92,424	0
Shewanella_denitrificans_OS217	91	2313	25045	240,069	0
Shewanella_frigidimarina_NCIMB_400	66	2286	26819	192,758	7
Shewanella_loihica_PV-4	68	2082	27260	181,814	5
Shewanella_oneidensis_MR-1	3954	3307	94195	5,561,459	442
Shewanella_putrefaciens_200	64	2200	25524	191,946	2
Shewanella_putrefaciens_CN-32	78	2338	29135	254,187	14
Shewanella_putrefaciens_W3-18-1	80	1975	18900	273,705	12
Shewanella_sp_ANA-3	62	2154	25870	145,474	4
Shewanella_sp_MR-4	65	2052	25724	195,547	6
Shewanella_sp_MR-7	64	2217	29196	178,776	16
Sinorhizobium_medicae	40	2557	17954	164,088	2
Sinorhizobium_meliloti_1021	137	2547	21631	581,060	2
Slackia_heliotrinireducens_DSM_20476	79	1645	22440	105,667	1
Stackebrandtia_nassauensis_DSM_44728	104	2158	22213	138,735	0
Sulfolobus_acidocaldarius_DSM_639	33	1380	16093	92,402	0
Synechococcus_sp_PCC7002	1050	2606	52469	1,842,452	1
Syntrophobacter_fumaroxidans	80	1893	18846	71,287	0
Thermobispora_bispora_DSM_43833	78	1470	9193	23,861	0
Thermosynechococcus_elongatus_BP-1	137	1266	8161	182,698	0
Thermosynechococcus_sp_NAK55	47	1106	9587	87,550	0
Thermotoga_maritima	281	1526	52050	544,795	6
Thiocapsa_marina_DSM_5653T	130	1632	8577	26,649	0
Verrucomicrobium_sp_TAV1	32	2288	15059	79,896	0
Verrucomicrobium_sp_TAV5	33	2116	12893	87,443	2
Xylanimonas_cellulosilytica_DSM_15894	200	2487	41090	319,030	0
Yersinia_enterocolitica	86	1622	11983	107,731	2
Yersinia_pestis_CO92	210	2302	33289	993,714	19
Yersinia_pestis_KIM	240	1371	11084	98,780	0
Yersinia_pestis_Pestoides_F	99	2153	22980	523,779	16
Yersinia_pseudotuberculosis_IP_32953	400	2151	24690	301,175	11
Yersinia_pseudotuberculosis_PB1_Plus	99	2029	20729	470,575	16
	35162	229,537	3,147,576	70,455,991	1951
Each organism is listed along with information about how many datasets, proteins, peptides and spectra were included in the Library creation. The number of false spectra identifications, as estimated by the decoy search strategy, is also included to help understand the quality of the data in the Library.					
